# Supplementary material for: Determining the drivers of global innovation under COVID-19: An FSQCA approach
Source: PLoS One. 2024 Feb 14;19(2):e0295403. doi: 10.1371/journal.pone.0295403 (PMC10866485; doi:10.1371/journal.pone.0295403)
Supplement: Attachment — Submitted filename: Response letter-10.27.docx [file pone.0295403.s002.docx]

**Dear Editors and Reviewers:**

Thank you to the editor and reviewers for their suggestions and comments. The comments really helped us see the big picture with the introduction.

***Reviewer 1***

Thanks for your encouragement and suggestions.

**Comments**

a) Abstract: somehow large, try to be more concise and with the present results from the article (remove the acronyms).

**Response:** We have revised the abstract according to your requirements to make it more concise and correspond to the current results of the article.

b) Introduction: NOVELTY + results (better explanation); try to reduce it to maximum 2 pages.

**Response:** We have made significant revisions to the introduction and rewritten it, mainly highlighting the highlights of our article and providing a better explanation of the results, resulting in a more refined content.

c)Literature review: better theoretical explanations (the actual explanation is quite general);

**Response:** We drew on the advantages of theoretical explanations and provided a more systematic introduction and explanation of practical explanations.

d)Section: 2.2 Construction of the research framework (very large 6 pages; try to reduce it to maximum 2 pages).

**Response:** We have abbreviated and refined the construction of the research framework according to the comments, reducing the word count by about two pages.

e) Section 4.1 Analysis of necessary conditions: quite statistical and inexpressive; try to improve the explanations.

**Response:** We have recognized our shortcomings and provided a deeper explanation of the necessary conditions analysis.

f) Discussions: At least 1 page (with the explanations for groups of countries; not general); separate from the conclusions.

**Response:** We separated the discussion from the conclusion and made it a separate part, providing an explanation of practical contributions, theoretical contributions, and limitations.

***Reviewer 2:***

Thanks for your encouragement and suggestions.

**Comments**

a) English is poor. Manuscript should be thoroughly examined and flow of ideas and use of language should be improved to be crystal clear.

**Response:** A native English-speaking professor help us to correct English language.

b) Problem definition should be articulated clearly.
**Response:** We have thoroughly reviewed the manuscript, improved the flow of ideas and the use of language, and made the definition of the problem clear.

c)Abstract: Flow of language is problematic. English should be checked.
**Response:** A native English-speaking professor help us to correct English language.

d)Covid-19 was a pandemic.
**Response:** At present, Covid-19 is no longer a pandemic. This is a mistake in our language expression, and we have made modifications to it.

1.Introduction
• Para-1-Senrence-1: Too deterministic.
• Para-1-Senrence-3: Abruptly innovation becomes a new topic with the provided definition. Narrative flow irritates reader.
• Para-1-Senrence-4: Too deterministic and assertive.
• Para-1: It is too long and jumping from one topic to another without proper conjunctions or transition elements to support the flow of narrative.
• Para2-Sentence-3: Topic is abruptly changes. Flow of narrative is problematic.
• Para-2: too long and contains many too assertive arguments when previous works are criticized. Probably the manuscript was developed during the pandemic. The expressions should be checked when “at present” type adverbs are used.
• Para-3 Sentence-1: “Black Swan Incident” simile should be properly explained. Why it cannot be considered “Black Swan Incident”?
• Para-3 Sentence-2: Very strong argument without any evidence. A reference can be useful to increase its persuasiveness.

**Response:** Due to the excessive number of issues in the introduction section, we have made significant modifications to the section, deleting some sentences that are too deterministic and lack theoretical basis, explaining why innovation has become a new topic, and adding appropriate conjunctions or transitional elements to support the fluency of the narrative. Like following:

By 2021, the COVID-19 pandemic had spread worldwide and altered international social and economic structures. How to manage the virus and when and how to open the economy again have become global issues. In response, governments were faced with the challenges of high-risk research and development (R&D), insufficient investment in innovation caused by the resulting economic downturn, and intensified trade competition due to intercountry control. To address these challenges, it is necessary to rationally allocate resources to promote innovation according to their own national conditions.

Different support systems and cooperative organizations related to industries form an innovative ecosystem that is both interdependent and symbiotic. The relationship between countries is heterogeneously synergetic rather than competitively oppositionist. The GII system vividly reflects the functions of various elements of the innovation ecosystem. It is a useful tool for evaluating innovation performance and can help guide policy-making. Actually, in view of the global innovation problem, academic circles have made many beneficial explorations. Many scholars have analyzed mainly the characteristics of innovation and development in a single country or region and made comparisons between these countries or regions. However, all the above studies explore the independent effects of single variables in accordance with deductive logic, ignoring the combined effect of conditions in the innovation ecosystem. Affected by the epidemic, the global investment policy environment is also full of uncertainties, it is not clear whether the deficiency of a single institutional element hinders innovation and how the conditional configuration systematically affects national innovation. Traditional regression analysis, which simply links income with innovation, faces practical problems. Since the start of the COVID-19 pandemic, research on how to improve global innovation has remained in the stage of theoretical construction, focusing only on the independent role of different factors at the levels of science, technology and organization, which limits the understanding of the synergistic pairing effect of multiple elements of innovation ecosystems to the difference in national innovation output. Based on the configuration perspective, this paper analyzes in-depth whether the configurations and single conditions that drive high innovation cause the bottleneck of national innovation.

The special innovation demand brought about by COVID-19 and the artificially created R&D barriers between countries in response to it have become the constraints of innovation in all countries. Actively responding to the epidemic influence and identifying experiences and lessons from it can provide an empirical basis for countries to complement each other's advantages, innovate collaboratively and overcome difficulties together when a pandemic occurs again. Therefore, against the background of COVID-19, based on the configuration perspective, combined with NCA and FSQCA, this study analyzes the necessary and sufficient causality and identifies the driving path for achieving high innovation. This study is helpful for broadening the perspective of national innovation-related research, deepening the understanding of the driving path and mechanism of national innovation against the background of the pandemic, and promoting the global process of actively responding to a pandemic with innovation.

2. LITERATURE REVIEW AND RESEARCH FRAMEWORK
2.1 Connotation and research progress of the national innovation index
Para-1: Flow of narrative is problematic. Assertive arguments such as “a lot of studies….” with two reference seems inadequate.
2.2 Construction of the research framework.
• There many assertive arguments without any support/evidence from the literature.
• Too long paragraphs with problematic narrative flow.
• Contains clumsily assembled arguments.
• Author(s) repeats citing a few certain studies such as Abi Younes et al., (2020) (cited 8 times through the text).
• Covid19 cannot be articulated as “current crises” in Fall 2023. These expressions should be calibrated.
• Johns Hopkins Center for Health Security should be written correctly.

**Response:** Firstly, we provided a detailed and detailed description of the literature review and research framework, but found it unnecessary. Therefore, we refined this section. Secondly, there are many arbitrary arguments in the article regarding the connotation and research progress of the National Innovation Index, such as "many studies...", so we have added some references. Finally, we will also replace the arguments for some clumsy combinations.

3. RESEARCH METHODS
3.1 Method of mixing NCA and QCA
The necessity of the methods employed in this study should be clearly stated. Reader cannot easily find out this.
3.2 Data sources.
Citations should be checked against references.
3.3 Variable measurement and calibration.
Calibration process seem vague. Clarity is required.
**Response:** Firstly, our research method, the hybrid method of NCA and QCA, is a major highlight of our article and should be explained in detail. Its necessity should also be clearly explained to help readers discover this. Secondly, we compared the data sources with the references and verified the citations. Finally, we also explained the calibration process. Like following:

Necessary condition analysis is indispensable in configuration analysis. NCA is a sufficient and direct method to identify the necessity of data, and the necessary condition can be expressed as "no X, no Y". In order to make up for the deficiency of QCA analysis method in necessity analysis, NCA analysis method is introduced on the basis of QCA analysis method for further inspection.

To demonstrate the technological progress and innovation capability of different countries more intuitively, Cornell University, the Institut Européen d’Administration des Affaires (INSEAD) and the World Intellectual Property Organization (WIPO) jointly published the Global Innovation Index (GII) 2021 edition, which provided indicators with which to measure innovation performance and rank the innovation ecosystems of 132 economies around the world, as 2021 was the year in which the whole world had to jointly deal with COVID-19 (Dutta et al., 2021).

In this study, direct calibration method is used to calibrate antecedent variables( ITT、	HCR、IFT、MS、BS)and result variables(Innovation). As the GII 2021 is a newly published measurement result, it lacks external and theoretical standards. Referring to previous studies three calibration points are set, namely, full membership, crossover and full non-membership of five conditional variables and one outcome variable, the original values of which are set to cover 95%, 50% and 5% of the data values, respectively (Table 1). The calibration of non-national innovation performance is realized by taking the non-set of high national innovation performance. The calibration results are shown in Table 1.

4. ANALYSIS RESULTS
4.1 Analysis of necessary conditions
Analyses conducted to explore the impact of different configuration should be clearly expressed.

**Response:** The analysis conducted to explore the impact of different configurations was expressed accordingly.

5. PERFORMANCE DIFFERENTATION PATH OF HIGH INCOME WITH
HIGH INNOVATION, LOW INCOME WITH HIGH INNOVATION, AND
HIGH INCOME WITH LOW INNOVATION
Full of hypothetical assertive statements without any evidence/reference.
**Response:** For arbitrary statements that are full of assumptions, we cite references for reference and argumentation.
6. CONCLUSIONS, DISCUSSION ANG FUTURE DIRECTION
6.1 Conclusions
Arguments asserting “Causality” seems questionable.
Analyses conducted seems inadequate to generalize any causation relation between variables. It’s adequacy /validity/reliability should be clearly explained with references to convince reader.
6.2 Theoretical contribution
Too deterministic and assertive statements. They should be checked and restated. Not understood clearly.
6.3 Practical contribution
Too deterministic and assertive statements. They should be checked and restated. Not understood clearly.
6.4 Limitations and directions for future research.
We do know why this study ignores international innovation interactions. Is it because of Covid19? This limitation should be explained.

**Response:** Firstly, citing references provides a clear explanation of the sufficiency, validity, and reliability of the conclusion to convince readers. Secondly, we have reviewed and reiterated the overly deterministic and arbitrary statements in our theoretical contributions. Once again, we have reviewed and reiterated the overly deterministic and arbitrary statements in our actual contributions. Finally, regarding limitations and future research directions, we explained the reasons for the decrease in international innovation interaction caused by Covid-19. Like following:

The innovation environment is the sum of internal and external factors that affect the development of innovation activities and the level of innovation performance, including innovation infrastructure, innovation policy system, innovation cultural atmosphere and so on. These factors are closely related and interact with each other, and jointly play a role in innovation activities and innovation performance. The outbreak and spread of COVID-19 epidemic not only affects people's health and lifestyle, but also affects the global innovation environment from many levels and fields, which is reflected in:

Reduce the supply of innovative infrastructure. Threatened by the virus epidemic, people have reduced all kinds of going out and communicating activities, and significantly reduced the scale of all kinds of production and innovation activities. At the same time, in order to curb the spread of the epidemic, local governments at all levels have continuously adopted and strengthened various preventive and control measures to limit crowd gathering and large-scale mobility. In this situation, the construction and operation of all kinds of innovation infrastructure are greatly restricted, and the supply is sharply reduced, which can’t meet the needs of all kinds of innovation subjects to carry out innovation activities. Not only is the rate of enterprises returning to work low, universities, research institutes, various intermediaries and platforms can't operate normally, and innovative elements such as related innovative resources, facilities and talents can't play their due roles, which makes the whole innovation system fall into inefficient operation or even stagnation to a great extent.

Increase the cost of innovation policy system. Affected by the COVID-19 epidemic, the government has shifted and adjusted its work focus from the strategic deployment at the national level to the specific path selection of local economic and social development. Many policies and systems to promote and guarantee innovation will inevitably give way to the medical field, which has significantly increased the operating costs of existing innovative policies and systems.

The epidemic situation has an unprecedented impact on innovation, and its negative impact on the entire innovation environment is also enormous.

Finally, the role statement of the sponsor, data availability, and competitive interest statement have all been improved and supplemented.

Once again, thank you very much for your comments and suggestions.

Thank you and best regards.

Yours sincerely,

Zhenxing Gong and Wag Yue.
