# Supplementary material for: Determining the drivers of global innovation under COVID-19: An FSQCA approach
Source: PLoS One. 2024 Feb 14;19(2):e0295403. doi: 10.1371/journal.pone.0295403 (PMC10866485; doi:10.1371/journal.pone.0295403)
Supplement: Attachment — Submitted filename: Response to Reviewers-11.18.docx [file pone.0295403.s003.docx]

***Dear Editors and Reviewers:***

Thank you to the editor and reviewers for their suggestions and comments. The comments really helped us see the big picture with the introduction.

***Reviewer 1***

Thank you very much for agreeing to publish my article in Plos One.

***Reviewer 2***

Thanks for your encouragement and suggestions.

**Comments**

• Why the epidemic period is focused? Why do we consider it "epidemic" in 2021? It was pandemic in 2021.

**Response:** Because the epidemic occurred at the end of 2019, 2020 was the initial stage of the epidemic, which mainly had a great impact on people's life safety, and 2021 was the most serious year, which began to have a major impact on all aspects of the country.

• Abbreviations should be checked. Deleted parts has gone with the long forms of them.

• Spelling issues still exist (e.g. entrie).

• There are problems on word selection in many cases.

**Response:** We checked the spelling and word selection carefully and made some corrections.

• Assertive hypothetical arguments with determinism still stay (e.g. comments about past innovation indexes).

• Deterministic and assertive arguments stay (e.g. “The relationship between income and innovation output is not simply proportional.”; “In the past, the complexity of causality between national innovation input and output had not been considered.”; “ITT is the main motivation for economic activities…….”; “Optimizing the business environment has a positive impact on effective corporate governance, business scope, competitive advantage and innovation ability”).

• Providing reference does not give you the right to use deterministic and assertive arguments. We are studying social science and all findings should be considered probabilistic. To be on the safe side it is always better to use elements such as “most of …”, “many …..”, “can be /is considered”.

**Response:** We have perfected the decisive and propositional arguments by collecting literature, and in order to pursue accuracy, we try to use words such as "most …", "many …" or "can/be considered".

• At the end of Introduction section, readers expect the aim of the study not implications. Robustness of the method should be discussed in the Methods section careful not in the introduction. “Do we propose a method?” or “Do we propose hypotheses regarding causality between variables?”

**Response:** We have modified the introduction to clarify the purpose of the study expected by readers, deleted the robustness of the method, and discussed it in the method part, being careful not to discuss it in the introduction. Like following:

1. INTRODUCTION

In 2021, an epidemic spread worldwide and altered international social and economic structures. The World Health Organization named it COVID-19. How to manage the virus and when and how to open the economy again have become global issues. In response, governments were faced with the challenges of high-risk research and development (R&D), insufficient investment in innovation caused by the resulting economic downturn, and intensified trade competition due to intercountry control. To address these challenges, it is necessary to rationally allocate resources to promote innovation according to their own national conditions.

Different support systems and cooperative organizations related to industries form an innovative ecosystem that is both interdependent and symbiotic. The relationship between countries is heterogeneously synergetic rather than competitively oppositionist. Actually, in view of the global innovation problem, academic circles have made many beneficial explorations. Many scholars have analyzed mainly the characteristics of innovation and development in a single country or region and made comparisons between these countries or regions. However, most of the above studies explore the independent effects of single variables in accordance with deductive logic, ignoring the combined effect of conditions in the innovation ecosystem. Affected by the epidemic, the global investment policy environment is also full of uncertainties, it is not clear whether the deficiency of a single institutional element hinders innovation and how the conditional configuration systematically affects national innovation. Traditional regression analysis, which simply links income with innovation, may faces practical problems. Since the start of the COVID-19 pandemic, many researches on how to improve global innovation had remained in the stage of theoretical construction, focusing only on the independent role of different factors at the levels of science, technology and organization, which limits the understanding of the synergistic pairing effect of multiple elements of innovation ecosystems to the difference in national innovation output. Based on the configuration perspective, this paper analyzes in-depth whether the configurations and single conditions that drive high innovation cause the bottleneck of national innovation.

The special innovation demand brought about by COVID-19 and the artificially created R&D barriers between countries in response to it have become the constraints of innovation in all countries. Actively responding to the epidemic influence and identifying experiences and lessons from it can provide an empirical basis for countries to complement each other's advantages, innovate collaboratively and overcome difficulties together when a pandemic occurs again. Therefore, against the background of COVID-19, based on the configuration perspective, combined with NCA and FSQCA, this study analyzes the necessary and sufficient causality and identifies the driving path for achieving high innovation.

3.1 The essentiality of using FSQCA and NCA

Several tenets suggest moving beyond multiple regression analysis to thinking and using algorithms.

First, based on correlation assumes that the variables are independent, the regression analysis method is suitable for exploring the net effect of a single explanatory variable on the explained variable. While FSQCA focuses on analyzing the multiple concurrent causal relationships formed by different combinations of causes and conditions, we can reveal the veil of how the causes and conditions interact together to affect the innovation outcomes (Pappas & Woodside,2021). The FSQCA method begins with holism, proceeds through an analysis of the complex causal relationships among many factors, and finally enables a cross-case comparative analysis to explore which configurations of conditional elements cause the expected results and which fail to lead to the expected result and result in other causal complexity problems. National innovation drives the combination of various factors to form different conditional configurations, and the complex influence on high national innovation output represents this type of problem, so the FSQCA method is especially suitable for this research.

Second, unlike correlation analysis, consistency is a test for sufficiency and not a test for sufficiency and necessity and FSQCA applies Boolean algebra to overcome the weakness of regression analysis and keeps the complex variable relationship aligned with further information about the research object, which mitigates the issue of missing variable deviation. The traditional quantitative method seeks to obtain the optimal solution of the result, while the qualitative comparative analysis method believes that the configuration leading to the result has equivalence, that is, the combination of multiple different conditions will produce the same result (Farivar et al.,2021). FSQCA can identify the sufficiency and necessity of the causes and conditions that cause the results, as well as the complementarity/substitution among different causes and conditions (Woodside,2013), and further deepen the understanding of different types of innovation input to innovation output.

Finally, the necessary condition refers to the condition needed for a specific result to occur. If this condition does not exist, then the corresponding result cannot be produced. NCA quantitatively demonstrates the antecedent level necessary to achieve a certain level of outcome variables by analyzing the effect size and bottleneck level of the antecedents (%).

• Idea flows are still problematic (e.g. At the national level, the gathering of knowledge workers leads to a region's emphasis on investment in education. The historically good positive cooperation among industry, universities, and -researchers has induced a number of research institutions to serve enterprises by increase their levels of R&D investment. Making it easier for innovators to take advantage of profit from their innovations through intellectual property protection. The level of motivation of innovators to generate new knowledge is insufficient, and the patent system provides a way of increasing the level of such motivations.--> This is located at the end of “2.2 Construction of the research framework.” Readers expect hypotheses at the end of this section.

**Response:** According to the requirements of the reviewers, we added some hypotheses leading to national innovation at the end of "2.2 Construction of Research Framework" to make readers better understand. Like following:

Therefore, we assume that ITT, HCR, IFT, MS and BS in the GII structure will have different degrees of influence on national innovation.

• Theoretical ideation part is confusing. I am not convinced about the necessity of the topic and method. “How the gap in the literature is located” and “how it is filled” aspects should be clearly stated combined with the definition of the problem and research question. At end of each theorization there should be clearly stated hypothesis articulating the expected causality between variables and/or combination of variables. Theorizations and arguments proposed by the authprs are not satisfactory.

**Response:** We have specially added a part in 3.Research methods to explain the necessity of the method. Like following:

3.1 The essentiality of using FSQCA and NCA

Several tenets suggest moving beyond multiple regression analysis to thinking and using algorithms.

First, based on correlation assumes that the variables are independent, the regression analysis method is suitable for exploring the net effect of a single explanatory variable on the explained variable. While FSQCA focuses on analyzing the multiple concurrent causal relationships formed by different combinations of causes and conditions, we can reveal the veil of how the causes and conditions interact together to affect the innovation outcomes (Pappas & Woodside,2021). The FSQCA method begins with holism, proceeds through an analysis of the complex causal relationships among many factors, and finally enables a cross-case comparative analysis to explore which configurations of conditional elements cause the expected results and which fail to lead to the expected result and result in other causal complexity problems. National innovation drives the combination of various factors to form different conditional configurations, and the complex influence on high national innovation output represents this type of problem, so the FSQCA method is especially suitable for this research.

Second, unlike correlation analysis, consistency is a test for sufficiency and not a test for sufficiency and necessity and FSQCA applies Boolean algebra to overcome the weakness of regression analysis and keeps the complex variable relationship aligned with further information about the research object, which mitigates the issue of missing variable deviation. The traditional quantitative method seeks to obtain the optimal solution of the result, while the qualitative comparative analysis method believes that the configuration leading to the result has equivalence, that is, the combination of multiple different conditions will produce the same result (Farivar et al.,2021). FSQCA can identify the sufficiency and necessity of the causes and conditions that cause the results, as well as the complementarity/substitution among different causes and conditions (Woodside,2013), and further deepen the understanding of different types of innovation input to innovation output.

Finally, the necessary condition refers to the condition needed for a specific result to occur. If this condition does not exist, then the corresponding result cannot be produced. NCA quantitatively demonstrates the antecedent level necessary to achieve a certain level of outcome variables by analyzing the effect size and bottleneck level of the antecedents (%).

• We cannot simply blame traditional regression analyses in finding causality. There are ways to test causality using the theoretical bases of regression. Better to use term “linear regression”.

**Response:** When looking for causality, we don't simply blame the traditional regression analysis, but explain its shortcomings and the advantages of our method. We also replaced "regression analysis" with the term "linear regression" according to the requirements of reviewers.

• I am not sure if the data set belongs to 2021 or 2020. This is info is not shared.

**Response:** In the article 3.3Date sources, we thought it explained our data source. Like following:

3.3 Data sources.

To demonstrate the technological progress and innovation capability of different countries more intuitively, Cornell University, the Institut Européen d’Administration des Affaires (INSEAD) and the World Intellectual Property Organization (WIPO) jointly published the Global Innovation Index (GII) 2021 edition, which provided indicators with which to measure innovation performance and rank the innovation ecosystems of 132 economies around the world, as 2021 was the year in which the whole world had to jointly deal with COVID-19 (Dutta et al., 2021).The country samples used in this analysis are the same as those referenced in the GII 2021 published in September 2021 (Cornell University, INSEAD, & WIPO, 2021)that reported the innovation of 132 countries worldwide. The choice of each group of countries by income (high-income and low-income countries) is determined using the World Bank income group classification (Cornell University, INSEAD, & WIPO, 2021).

• The method employed is a robust and promising for cross-sectional data sets in social sciences. However, there should be more explanation about the method. "What are the theoretical bases?" "How these figures are calculated?" Is there a software used for them? "Are they calculated manually?". Besides, implications could be better stated and discussed.

**Response:** In view of our data analysis software, we have carried on the concrete explanation in the article. Like following: NCA is a method to analyze data by using R software.

Once again, thank you very much for your comments and suggestions.

Thank you and best regards.

Yours sincerely,

Zhenxing Gong and Wag Yue.
